# Uptake of Trace Elements in the Water Fern *Azolla filiculoides* after Short-Term Application of Chestnut Wood Distillate (Pyroligneous Acid)

**DOI:** 10.3390/plants9091179

**Published:** 2020-09-11

**Authors:** Zuzana Fačkovcová, Andrea Vannini, Fabrizio Monaci, Martina Grattacaso, Luca Paoli, Stefano Loppi

**Affiliations:** 1 Department of Life Sciences, University of Siena, via Pier Andrea Mattioli 4, I-53100 Siena, Italy; zuzana.fackovcova@unisi.it (Z.F.); andrea.vannini@unisi.it (A.V.); fabrizio.monaci@unisi.it (F.M.); grattacaso@student.unisi.it (M.G.); 2 Institute of Botany, Plant Science and Biodiversity Centre, Slovak Academy of Sciences, Dúbravská cesta 9, SK-84523 Bratislava, Slovakia; 3 Department of Biology, University of Pisa, via Ghini 13, I-56126 Pisa, Italy; luca.paoli@unipi.it

**Keywords:** aquatic biota, biocontrol, ecotoxicology, wood vinegar, heavy metals

## Abstract

Treatments of crops with additives to increase their productivity may pose environmental risks and induce negative effects also on non-target organisms. In this study, we investigated the potential effect of chestnut wood distillate (pyroligneous acid) used in agriculture, on the accumulation of trace elements in aquatic plants. As a model species, the common water fern *Azolla filiculoides* Lam. was selected, being often used also in phytoremediation processes. The content of selected elements of toxicological concern (As, Ba, Cd, Cu, Fe, Mn, Ni, Pb, Zn) was assessed in the fern after short-term treatments (1–3 days) over a range of wood distillate concentrations 1:300 (3.33 mL/L), 1:500 (2.00 mL/L), 1:700 (1.43 mL/L). A statistically significant accumulation of Cd, Cu, Mn, Pb, Zn (1:700) and Pb (1:300) was recorded after three days of incubation, despite the concentrations remained overall low. Using treatment vs. control ratios, a trend of increasing temporal uptake was detected for As, Ba, Fe, Mn, Pb (1:700); Mn, Pb (1:500), and only Pb at 1:300. The results suggested that, under the experimental conditions, element uptake is positively influenced by time and negatively by increasing concentrations of wood distillate, likely due to the acidification of the medium. On the whole, the element concentrations measured in *A. filiculoides* were low and did not pose any toxicological concern.

## 1. Introduction

The application of synthetic pesticides and fertilizers is a common practice to increase crop productivity all over the world. However, undesirable side effects on biota and the environment caused by their excessive use are widely recognized, including ecosystem imbalance, environmental contamination and damages to non-target organisms [1,2,3]. These and other negative aspects led to the development of organic agriculture with environmentally-friendly practices, promoting among the appropriate regulatory frameworks a reduced use of synthetic pesticides, herbicides and fertilizers. These practices are expected to mitigate climate change, desertification, to preserve biodiversity and produce significant social, economic and environmental benefits [4,5]. Furthermore, they are promoted in the framework of the Sustainable Development Goals (SDGs) to be reached in 2030 [6] and included in policy-driven and regulatory documents of several countries [7,8,9,10]. Therefore, the development of alternative natural products that could substitute synthetic chemicals and minimize environmental risk whilst improving agricultural production is currently of great concern.

One of such alternative products is wood distillate, a by-product of bioenergy and biofuel production. In this process, the plant biomass is converted by controlled pyrolysis, gasification and carbonization into gaseous, liquid and solid biofuels of commercial value, such as biochar, syngas, vegetable tar, and wood distillate [11,12]. Since the biomass for the combustion originates from wood management residues, this bio-production also enhances a sustainable use of biomass as a source of renewable energy [13]. This is currently an important issue, due to ineffective biomass waste disposal, utilisation and management in many countries [14,15].

Wood distillate (also called pyroligneous acid, wood vinegar, liquid wood, liquid smoke, pyrolysis oil, bio-oil, or wood oil) is extracted by vapour distillation over different temperature gradients in absence of oxygen. It is produced without the addition of synthetic chemicals and during the pyrolysis process, only the physiological water occurring in the sapwood is used. The extract passes through natural filters and is free of residues. According to the characteristics of the technological process used (e.g., biomass origin, moisture content of biomass, particle size, heating rate, temperature, residence time), wood distillate may feature slightly different properties which can subsequently lead to fine differences in its effectiveness [16,17,18,19,20]. Wood distillate can be applied to promote plant development and increase crop productivity and quality [21]. Its chemical composition is quite complex, but is mainly characterized by high contents of acetic acid, phenols and esters, which stimulate antioxidant [22,23,24] and antimicrobial properties [25,26] and enhance plant growth [27,28,29].

However, once applied, wood distillate may be leached and potentially affect the surrounding environment. Therefore, the associated ecological risk should be assessed also on non-target organisms, as defined by international regulations for this type of product [30]. Presently, only few studies focused on the potential ecotoxicological effects of wood distillate on soil fauna, microbes, plants [31], mosses and lichens [32]. Since the chemical compounds applied in the terrestrial ecosystems are often found also in aquatic environments [33,34,35,36], it is important to assess their effects also on the aquatic biota. The toxicity of birch–tar–oil (BTO) on aquatic fauna, flora and bacteria has been tested by Hagner et al. [37], in the framework of research aimed at testing the effect of BTO as molluscicide, and thus at very high concentrations (up to 4900 mg/L). However, to the best of our knowledge, the effects of wood distillate on non-target aquatic ferns at ecologically relevant concentrations have not been studied, yet.

The present article deals with one of the potential effects, namely the uptake of trace elements of toxicological relevance, in the aquatic biota after wood distillate application. As a model species, we selected the common floating water fern *Azolla filiculoides* Lam.: the species has a worldwide distribution and is characterized by a symbiotic association with the nitrogen-fixing cyanobacterium *Anabaena azollae* Stras. Thanks to this nitrogen-fixing ability, it can be used also to stimulate the growth of crops in water (e.g., rice) [38]. Furthermore, thanks to its ability in removing heavy metals and other contaminants (e.g., azo dyes, hydrazine) from the aquatic environment, this species has been studied for potential applications in phytoremediation processes [39,40,41,42,43]. In addition, it has been employed as a non-target organism to detect the potential effects of pharmaceuticals released in the aquatic environment [44,45].

In an earlier article [32], the effects of wood distillate on the vitality and uptake capacity were tested in mosses and lichens as non-target organisms for the first time. The present study was carried out to reveal whether the application of different concentrations of wood distillate may significantly alter the chemical composition of the aquatic fern *A. filiculoides*.

## 2. Results

Trace elements measured in *A. filiculoides* incubated in wood distillate solutions of different concentrations are summarized in Table 1. The chemical content of control samples varied over time and resulted in the leaching of several elements after 3 days (As, Ba, Fe, Mn, Pb). On the whole, Cd, Cu and Zn remained stable and Ni showed fluctuations (Table 1). Compared with control values, after the first day of incubation, a significant uptake (*p* < 0.05) was observed for Cd (1:700) and Ni (1:500). After the second day, this pattern was detected for Cd (1:700) and Zn (1:300), and after the third day for Cd, Cu, Mn, Pb and Zn (1:700) and Pb at 1:300. Decreasing contents (*p* < 0.05) of trace elements (leaching) after wood distillate treatments were observed after the first day only for As (1:700), Ba and Mn (all treatments) and after the second day for Ba and Mn (1:300).

**Figure 1:**
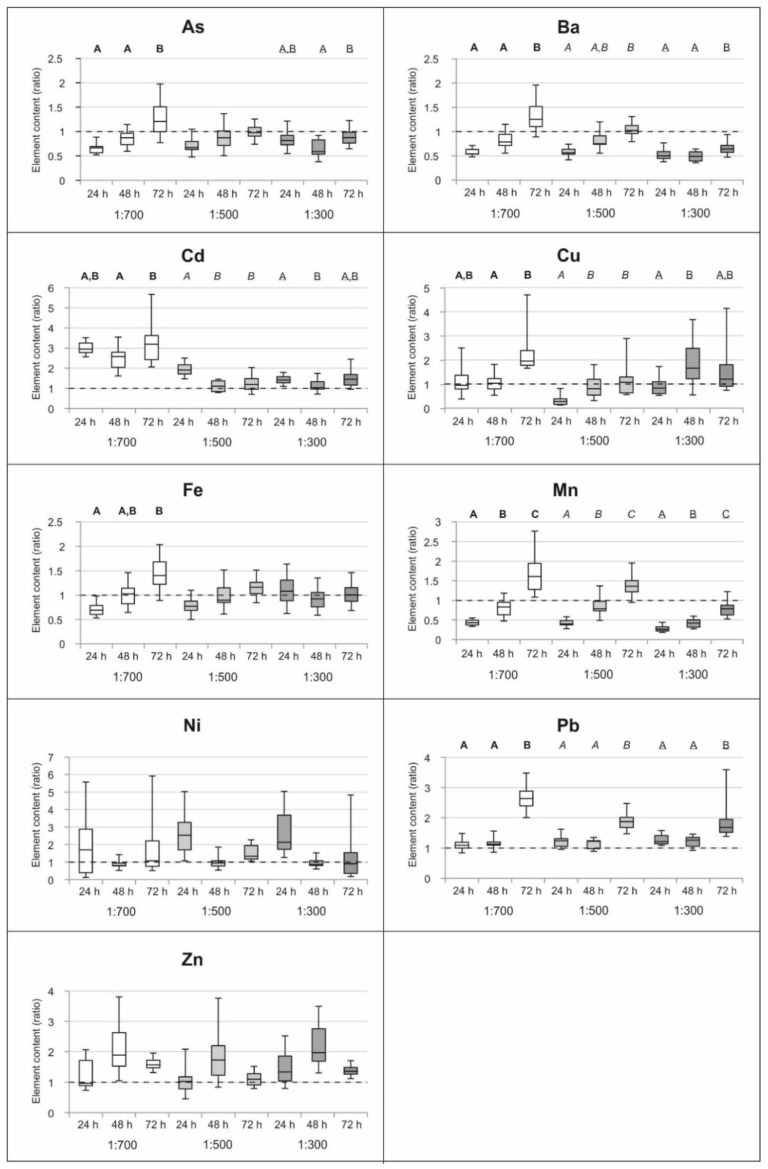
Expression of trace elements content in *Azolla filiculoides* (in terms of median, quartiles, min and max of ratios–normalized values to the control) treated with wood distillate solutions at 1:700, 1:500, 1:300 after three incubation periods (24 h, 48 h, 72 h) (*N* = 3). Statistically significant (*p* < 0.05) differences between the treatments along time are indicated by different capital letters (bold for 1:700, italic for 1:500, underlined for 1:300).

Accounting for the variation in control samples, to compare the data over time, we normalized the chemical content of treated samples to their respective control and obtained normalized ratios to be compared. The results are shown in Figure 1. Any significant trend of uptake or release over time was observed for Ni and Zn. On the other hand, the lowest concentrations of wood distillate (1:700) promoted a significant uptake of As, Ba, Fe, Mn and Pb over time and similarly, incubation with 1:500 solutions induced the uptake of Mn and Pb at the end of the treatments. Fluctuations at the beginning were observed for Ba, Cd and Cu (1:500); however, their final ratios around one suggested a stable content (no uptake or release). Wood distillate at the highest tested concentration (1:300) promoted an increasing ratio over time for Ba and Mn (however still < 1 indicating loss of elements). A weakly increasing trend with significant uptake after the third day was observed in the case of Pb.

## 3. Discussion

In our experiment, the trace element content of the water fern *A. filiculoides* treated upon a range of wood distillate concentrations highlighted a statistically significant uptake of Cd, Cu, Mn, Pb, Zn (1:700) and Pb (1:300) after three days of incubation, despite the fact that the concentrations remained low overall. Although the element content of the fern varies depending on the surrounding environment, our samples overall reflected a condition comparable to non-contaminated samples, even after the treatments [46,47].

Noteworthily, the chemical content in control samples varied in time and resulted in the leaching of some elements, namely As, Ba, Fe, Mn, Pb, after three days of incubation, likely reflecting a continuously changing equilibrium of the chemical content of the fern incubated with mineral water. It is possible that the prolonged contact between control samples and their solution (containing only mineral water) could have determined the partial leaching of some ions, occurring especially after three days.

Since the composition of each pyroligneous acid can differ according to the feedstock and the technological processes used for the production, direct comparisons of the results with literature data should be performed with caution. In our case, it was possible to compare the results about potential bioaccumulation in the water fern with other cryptogams (mosses and lichens), which have a high ability to take up nutrients and contaminants all over their surfaces, being thus sensitive and useful bioindicators of environmental contamination [32]. Hence, a comparison of similar wood distillate treatments is displayed in Table 2. For the comparison, we selected the final time of incubation to reflect the maximal uptake/releasing of elements considering the different nature of the selected cryptogams.

Treatments of the lichen *Xanthoria parietina* and the moss *Hypnum cupressiforme* with the same type and concentrations of chestnut wood distillate induced only low bioaccumulation: the uptake of the same toxic elements remained low overall and did not show a clear pattern with increasing concentrations of wood distillate [32]. In general, it seems that wood distillate influenced differently the chemical content of trace elements in different cryptogams. In the lichen, a significant uptake was revealed for Ba, Fe, Ni and Pb at mostly moderate wood distillate concentrations, in the moss only for Pb. Significant uptake of Cd, Cu and Zn was observed solely in the fern (mostly at the lowest wood distillate concentration). On the other hand, Pb uptake occurred in all organisms, irrespective of the concentration of the treating solutions.

The genus *Azolla* has a remarkable capacity to concentrate elements, including toxic heavy metals, in its biomass [48]. The ability of *A. filiculoides* to accumulate heavy metals from treating solutions has been highlighted in several studies. For example, treatments of *A. filiculoides* with solutions containing Ni, Cd and Pb- cations (5, 10 and 25 mg/L) showed an increased removal efficiency (from 40 up to 70%) by increasing the contact time up to 10 days [40]. Khosravi et al. [49] revealed that living samples of *A. filiculoides* were able to remove Pb^2+^, Cd^2+^, Ni^2+^ and Zn^2+^ from aqueous solutions enriched by 4 mg/L of metal ions and their presence caused up to 42% inhibition of biomass growth. Similarly, living samples of *A. filiculoides* treated with toxic Cr solutions up to 10 mg/L were able to produce only 30–70% biomass as compared to controls [50]. An interesting uptake capacity (following different kinetics) was also reported for samples in the dry (dead) state [50,51], further supporting the use of this fern for treating metal-contaminated wastewaters.

Fronds of fresh *A. filiculoides* were also tested for their potential uptake capacity in wastewater remediation and showed high removal efficiency for Fe (92%) and Al (96%), while for other elements, such as Cr, the removal was <10% [45]. The fronds were treated up to 8 days with individual solutions (heavy metals at 5 mg/L) characterized by pH in the range 6.9–7.2. The treatments also highlighted low toxicity under the experimental conditions, and even a stimulatory effect on the fern growth for treatments with Al-enriched solutions [45]. A comparison with our results suggests that the uptake could have been affected by pH, with wood distillate solutions being characterized by lower pH values (5.50 at 1:300) than controls (7.60). Hence, increasing concentrations of acetic acid in the solutions could have limited the efficiency of root uptake. In fact, pH-dependent responses were already reported in *A. filiculoides* during metal uptake [52,53]). Under laboratory conditions, the fern shows higher growth rates and nitrogenase activity over a wide range of pH values, with the best performances in the interval 5–7 and a deep decrease at lower values [54].

Optimal pH values for adsorption of metal ions Pb^2+^, Cd^2+^, Cu^2+^ and Zn^2+^ by *A. filiculoides* were determined as 5.5, 6, 5.5 and 6.7, respectively [55]. Ahmady-Asbchin et al. [53] pointed out that the samples of *A. filiculoides* treated with Ni solutions revealed the highest biosorption capacity at pH 7.8. The significant decrease of biosorption levels in the fern by lowering pH was attributed to the competition between protons and metal ions for the same binding sites, in which at low pH, metal ions are not successful [53]. Similarly, the competition for binding sites represents a limiting factor for the uptake of heavy metal ions also in mosses and lichens [56,57]. A limited uptake of heavy metals from wood distillate observed in the lichen *X. parietina* and the moss *H. cupressiforme* [32] could have been influenced by a lower pH of the treating solutions (3.6–3.8, in deionized water) and a shorter contact time (24 h), respect to the actual treatments.

Most heavy metal uptake in living *A. filiculoides* occurs via roots. Samples treated for 3–7 days in a nutrient medium containing 8–15 ppm of several heavy metals (Cd, Cr, Cu, Ni and Zn) showed metal concentrations in the roots 2–5 times higher than in the shoots and the contemporary leakage of several macroelements from the plant [42]. The accumulation of high levels of some heavy metals in roots vs. relatively small amounts in shoots may reflect scarce mobility and translocation in other parts of the plant [42]. Element uptake via roots resulted in the localization of heavy metals (e.g., Cu and Cd) in the inner epidermis, cortex and bundle cell walls of the root [58], while for other elements (e.g., Pb), after root uptake, translocation and storage were observed in the vacuoles of the mesophyll cells [59]. In this sense, element uptake can be limited when roots are damaged or detached. This seems the case of our samples after three days of wood distillate treatment: empirical observation on the amount of *A. filiculoides* roots detached suggested a relationship between the concentration of wood distillate (or lower pH) in the treating solution and the damage endured by the roots (Supplementary Materials Figure S1). This could also explain, at least in part, a limited uptake than expected at the highest wood distillate concentration (1:300), as seen from Table 1. In fact, a significant uptake was detected only in the case of Pb and Zn, whereas at the lowest concentrations (1:700), the uptake occurred for Cd, Cu, Mn, Pb, Zn. These patterns appeared also considering temporal differences (Figure 1), where at 1:700, the uptake was much more evident. However, on the whole, all measured concentrations in *A. filiculoides* are very low and do not reflect values of ecotoxicological concern.

Furthermore, treatments with the same wood distillate concentrations in the lichen *X. parietina* did not induce physiological changes, while in the moss *H. cupressiforme*, they only caused a modest variation of photosynthetic parameters and a progressive increase of antioxidant activity according to the dose supplied [32]. The studies by Hagner et al. [37] on birch–tar–oil (BTO) effects on aquatic organisms (sediment invertebrates, fishes, pond snails, aquatic plants, unicellular green algae, fluorescent bacteria) provided interesting evidence about their sensitivity to this type of pyroligneous acid, and also suggested that it did not pose a severe hazard to the aquatic biota. However, the IC50 values reported by these authors were much higher than the concentrations tested in the present study. As an example, the IC50 value of BTO for the aquatic plant *Lemna minor* after 7 days of treatment was 231 mg/L for the roots and 229 mg/L for the fronds, while up to 137 mg/L a stimulating effect was noted. These values have to be compared with the ca. 3.5 mg/L tested at the highest chestnut wood distillate concentration used in the present study. In this sense, as very little is known about the interactions of wood distillate with aquatic (and in general non-target) organisms, further studies are needed, particularly to investigate possible chronic long-term toxic effects of ecologically relevant wood distillate concentrations.

## 4. Materials and Methods

### 4.1. Sample Collection

About four m^2^ of the floating water fern *Azolla filiculoides* (fully covered surface) were collected at the end of September 2019 from an artificial pond at the Botanical Garden of the University of Siena, Italy (WGS84: N43.312996°, E11.330026°). After collection, the samples were carefully cleaned from inorganic materials attached to the roots, gently washed with mineral water, then placed into glass jars with mineral water and left to acclimate at ambient conditions for two days. The plant material from this experimental site has been already selected for previous studies [44].

### 4.2. Characteristics of Wood Distillate Used

The selected chestnut (*Castanea sativa* Mill.) wood distillate (“Distillato di Legno BioDea” by Esperia s.r.l.) is characterized by pH 3.5–4.5, density 1.05 kg/L, acetic acid content 2.0–2.3%, content of phenols 2.90–3.02 g/kg and polyphenols 23–26 g/kg [60]. The trace element content of pure wood distillate was determined by inductively coupled plasma mass spectrometry (ICP-MS Perkin Elmer–Sciex, Elan 6100), resulting in the following concentrations (in µg/L): As–7, Ba–63, Cd–12, Cu–426, Fe–32,600, Ni–54, Pb–231, Zn–3847 [32]. In the present work, also Mn was considered and the concentration was 441 µg/L.

### 4.3. Application of Wood Distillate

After acclimation, the samples of the water fern *A. filiculoides* were transferred to solutions of wood distillate diluted with mineral water at concentrations 1:300 (3.33 mL/L), 1:500 (2.00 mL/L), and 1:700 (1.43 mL/L) and in mineral water only (control; characteristics available in rp-2013-12953, rapporto di prova, acqua minerale—naturale viva, s.i.a.mi. Spa). Three glass jars with a volume of 700 mL and a surface of 320 cm^2^ were prepared for each treatment. The samples were incubated for 24 h, 48 h and 72 h. The experiment was run at ambient temperature and light conditions (photoperiod ca. 12 h). After incubation, samples were left to dry in a climatic chamber (T = 16 °C, RH = 50% light = 40 μmol m^−2^ s^−1^ photons PAR) for 24 h and then stored at −20 °C until analyses.

The tested concentrations of wood distillate correspond to the recommended usage range for application in agriculture as biostimulant [60]. The pH of the solutions was not adjusted to a defined value as this is not standard practice in real use, and the single values (control, 1:700, 1:500, 1:300) were 7.60, 6.95, 6.40 and 5.50, respectively.

### 4.4. Trace Element Analyses

The samples (150 mg of dry weight material) were mineralized with a mixture of 3 mL of 70% HNO_3_, 0.2 mL of 60% HF and 0.5 mL of 30% H_2_O_2_ in a microwave digestion system (Milestone Ethos 900) at 280 °C and 55 bar. The content of nine elements (As, Ba, Cd, Cu, Fe, Mn, Ni, Pb, Zn) was determined by inductively coupled plasma mass spectrometry (ICP-MS Perkin Elmer–Sciex, Elan 6100) and expressed on a dry weight basis (μg/g dw). The analytical quality was checked with the certified Standard Reference Material NCS DC73350 ‘Leaves of Poplar’ (recoveries in the range 93–105%). The analytical precision was 87% for As and ≥93% for all remaining elements. For each treatment and time, three replicates were measured.

### 4.5. Statistics

In order to check whether significant uptake or leaching occurred, given the limited dataset, statistically significant differences between treatments and controls were checked using a permutation test based on 1000 randomized runs. The *p*-values below 0.1 were subsequently checked by repeated permutation test with 100,000 randomized runs, as suggested by Rice and Lumley [61]. Since significant differences were detected among the controls during the experiment, values were normalized to the respective controls and analyzed to test the significance of uptake or release within each treatment over time. For this purpose, the Wilcoxon W signed rank test was used. Outliers were checked by the Tukey test. All analyses were run using the R software [62].

## 5. Conclusions

The short-term application of chestnut wood distillate solutions to the aquatic fern *A. filiculoides* promoted variations in its trace elements content, which were positively influenced by time (increasing within three days) and negatively by concentrations of wood distillate solutions (decreasing from 1:700 to 1:300). However, although statistically significant changes were observed, the content of trace elements was very low and did not exceed the values of non-contaminated *A. filiculoides* samples, suggesting that chestnut wood distillate applications in recommended usage doses do not pose a significant risk in terms of heavy metal accumulation. This study provides initial insights into the short-term effects of this specific wood distillate on the aquatic biota. Since, in some cases, repeated treatments by wood distillate on agricultural crops are suggested to increase their productivity, the effects of long-term treatments need to be investigated.

## Figures and Tables

**Table 1:** Mean concentrations (μg/g dw ± standard deviation) of trace elements detected in the aquatic fern *Azolla filiculoides* after the first, second and third day of incubation in mineral water (control) and 1:700, 1:500 and 1:300 wood distillate solutions (N = 3). For each day, statistically significant (*p* < 0.05) differences between treatments are indicated by different letters. Asterisks mark control values of the second and third day which are significantly different from the control values of the first day. For each day, arrows summarize the overall pattern (↑ uptake, ↓ release, – not relevant) of significant changes respect to their control.

Treatment	Trace Element Concentrations (Mean ± SD)								
1st day	As	Ba	Cd	Cu	Fe	Mn	Ni	Pb	Zn
Control	1.458 ± 0.224 a	90.1 ± 12.6 a	0.059 ± 0.007 a	2.14 ± 0.73	1505 ± 260 ab	2428 ± 469 a	1.84 ± 0.90 a	1.19 ± 0.16	18.2 ± 7.1
1:700	0.929 ± 0.118 b	50.9 ± 3.5 b	0.175 ± 0.007 b	2.23 ± 1.11	1052 ± 142 a	1024 ± 54 b	2.92 ± 2.45 ab	1.30 ± 0.20	19.6 ± 2.2
1:500	1.028 ± 0.251 ab	51.5 ± 7.8 b	0.113 ± 0.017 ab	0.62 ± 0.39	1144 ± 246 ab	1009 ± 192 b	4.84 ± 1.93 b	1.43 ± 0.20	17.7 ± 5.7
1:300	1.208 ± 0.304 ab	47.6 ± 10.6 b	0.083 ± 0.010 ab	1.77 ± 0.44	1588 ± 466 b	677 ± 174 b	4.05 ± 0.65 ab	1.50 ± 0.08	24.1 ± 3.7
**2nd DAY**									
Control	1.140 ± 0.280	75.7 ± 18.5 a	0.084 ± 0.023 a	1.95 ± 0.76	1224 ± 355	1827 ± 602 a	5.18 ± 1.50 *	1.07 ± 0.18	13.1 ± 5.6 a
1:700	0.944 ± 0.107	59.3 ± 7.9 ab	0.203 ± 0.024 b	1.89 ± 0.39	1184 ± 144	1376 ± 197 ab	4.35 ± 0.96	1.22 ± 0.20	24.2 ± 4.4 ab
1:500	0.983 ± 0.272	59.4 ± 10.0 ab	0.090 ± 0.003 a	1.55 ± 0.69	1133 ± 209	1443 ± 332 ab	4.84 ± 1.55	1.22 ± 0.07	22.1 ± 6.3 ab
1:300	0.728 ± 0.160	35.8 ± 1.8 b	0.091 ± 0.017 a	3.49 ± 1.70	1079 ± 141	717 ± 65 b	4.65 ± 0.82	1.27 ± 0.09	28.0 ± 4.7 b
**3rd day**									
Control	0.859 ± 0.119 *	59.7 ± 7.7 * ab	0.089 ± 0.020 a	3.54 ± 1.31 a	840 ± 140 *	1242 ± 230 a *	2.80 ± 1.69	0.58 ± 0.08 a *	22.9 ± 2.6 a
1:700	1.085 ± 0.355	78.7 ± 20.9 a	0.282 ± 0.085 b	8.21 ± 1.24 b	1191 ± 271	2160 ± 775 b	3.14 ± 0.90	1.50 ± 0.20 b	36.3 ± 3.1 b
1:500	0.843 ± 0.136	61.4 ± 9.5 ab	0.108 ± 0.040 a	3.58 ± 1.52 a	958 ± 152	1679 ± 377 ab	4.95 ± 0.24	1.07 ± 0.17 ab	25.3 ± 6.5 a
1:300	0.754 ± 0.116	38.9 ± 7.1 b	0.126 ± 0.026 a	4.69 ± 1.46 a	846 ± 146	946 ± 192 a	2.84 ± 2.32	1.27 ± 0.66 b	31.2 ± 2.0 ab
**Summary:**	↓ – –	↓ ↓ –	↑ ↑ ↑	– – ↑	– – –	↓ ↓ ↑	↑ – –	– – ↑	– ↑ ↑

**Table 2:** Variations of trace elements content (↑ uptake, ↓ release, N.A not analyzed) in different organisms after the application of wood distillate (at 1:700, 1:500 and 1:300). Cumulative summary based on the data obtained at the end of the experiments – 72 h of exposure for the fern (this study) and 24 h for moss and lichen [32].

	Trace Element Uptake or Releasing								
Species	As	Ba	Cd	Cu	Fe	Mn	Ni	Pb	Zn
Fern			↑ 1:700	↑ 1:700		↑ 1:700		↑ 1:700, 1:300	↑ 1:700
Moss	↓ 1:700, 1:500	↓ 1:300				N.A		↑ 1:700, 1:500	
Lichen		↑ 1:500			↑ 1:700, 1:500	N.A	↑ 1:500	↑ 1:500, 1:300	

## Supplementary Materials

The following are available online at https://www.mdpi.com/2223-7747/9/9/1179/s1, Figure S1: Azolla *filiculoides* roots detached in a selection of control, 1:700, 1:500, and 1:300 wood distillate solutions after three days of treatment.
