# Supplementary figures and images for: Uptake of Trace Elements in the Water Fern Azolla filiculoides after Short-Term Application of Chestnut Wood Distillate (Pyroligneous Acid)
Source: Plants (Basel). 2020 Sep 11;9(9):1179. doi: 10.3390/plants9091179 (PMC7569869; doi:10.3390/plants9091179)

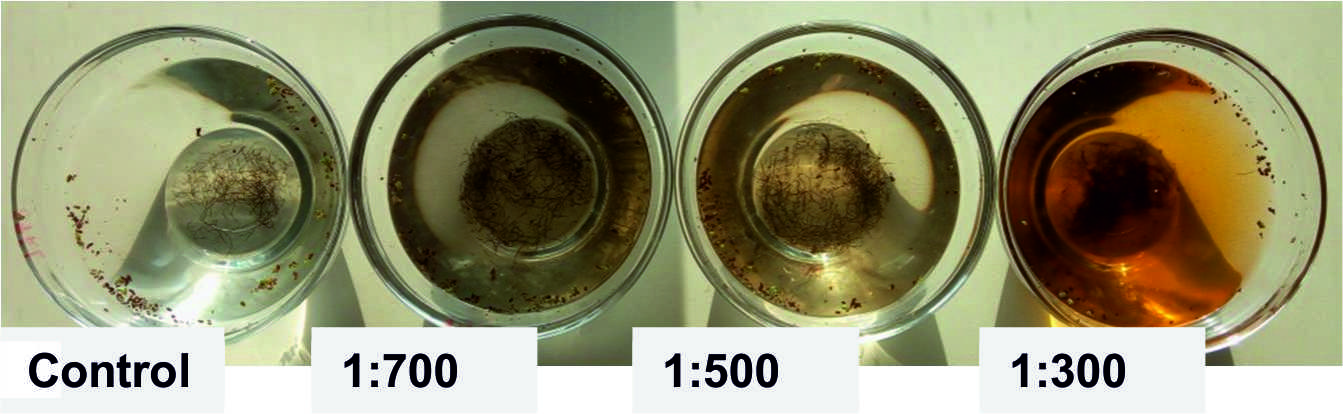

Supplement: Supplementary file 1 [file plants-09-01179-s001.zip › plants-927087-supplementary.jpg]
